# Pancreatic kaposiform hemangioendothelioma complicated by Kasabach–Merritt phenomenon: A rare entity

**DOI:** 10.4102/sajr.v23i1.1760

**Published:** 2019-08-19

**Authors:** Denny Mathew, Nasreen Mahomed

**Affiliations:** 1 Department of Diagnostic Radiology, University of the Witwatersrand, Johannesburg, South Africa; 2 Department of Radiology, Rahima Moosa Mother and Child Hospital, University of the Witwatersrand, Johannesburg, South Africa; 3 South African Society of Paediatric Imaging (SASPI), Cresta, South Africa

**Keywords:** Radiology, Paediatrics, Paediatric Surgery, Paediatric Radiology, Pancreas Vascular Tumours

## Abstract

Primary pancreatic tumours are a rare and unusual entity in children. In this article, we present the case of an 8-month-old girl who presented with obstructive jaundice. The differential diagnosis based on imaging studies was that of a pancreatic vascular neoplasm; however, with the laboratory evidence of Kasabach–Merritt phenomenon (KMP), this prompted the diagnosis of pancreatic kaposiform hemangioendothelioma. A core biopsy of the pancreatic mass was taken at laparotomy and confirmed this diagnosis. The pancreas is an exceedingly rare site of occurrence for this tumour, with only nine cases being published to date. The clinical, biochemical, imaging and pathological findings are discussed to highlight a rare and potentially life-threatening vascular tumour.

## Introduction

Kaposiform hemangioendothelioma (KHE) is a rare, high-flow vascular tumour of infancy or early childhood that was first described by Zuckerberg et al. in 1993.^1,2^ Kaposiform hemangioendothelioma is a locally aggressive tumour that may infiltrate the surrounding tissues and is usually of cutaneous origin from the extremities, torso body wall and cervico-facial regions.^1^ Retroperitoneal, mediastinal and visceral organ involvement are rare.^1^ The incidence of KHE based on the cases observed at a large referral centre has been estimated to be 0.07/100 000 children per year, while retroperitoneal KHE was reported in no more than 30 cases in the English literature.^1,3^ The pancreas is an exceedingly rare site of occurrence for this tumour, with only nine cases being published to date.^4^ We present the case of a pancreatic KHE involving the head of the pancreas.

## Case presentation

An 8-month-old girl presented with signs and symptoms of obstructive jaundice, with an associated history of progressive abdominal distension. Serology revealed elevated liver ductal and transaminase enzymes with conjugated hyperbilirubinaemia, normocytic anaemia, thrombocytopenia and coagulation abnormalities. The liver function test results were as follows: total bilirubin 92 µmol/L (normal range 0–21), conjugated bilirubin 86 µmol/L (normal range 0–6), gamma-glutamyltransferase (GGT) 967 U/L (normal range 1–39), alkaline phosphatase (ALP) 1117 U/L (normal range 124–341), alanine transaminase (ALT) 134 U/L (normal range 3–30) and aspartate transaminase (AST) 99 U/L (normal range 0–79). The haemoglobin level was 9.0 g/dL (normal range 10.5–13.7), mean corpuscular volume (MCV) 84.4 fl (normal range 70.0–86.0) and platelets 21 ×10^9^/L (normal range 180–440).

The abdominal ultrasound (US) demonstrated a bulky, homogeneous, soft tissue mass in the head of the pancreas that was isoechoeic to the remainder of the pancreatic gland, with associated findings of dilated intrahepatic bile ducts and ascites. Internal flow was demonstrated within this lesion on colour flow Doppler assessment.

**FIGURE 1:**
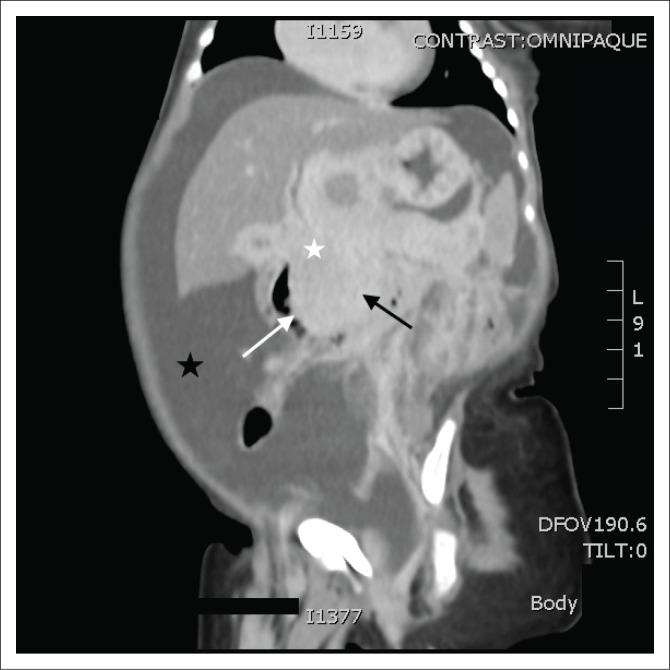
Coronal post-contrast computed tomography image demonstrating an ill-defined, enhancing mass in the head of the pancreas (black arrow), with a poor plane of separation from the D2 segment of the duodenum (white arrow) and peripancreatic extension to the porta hepatis (white star). Secondary findings of ascites (black star).

**FIGURE 2:**
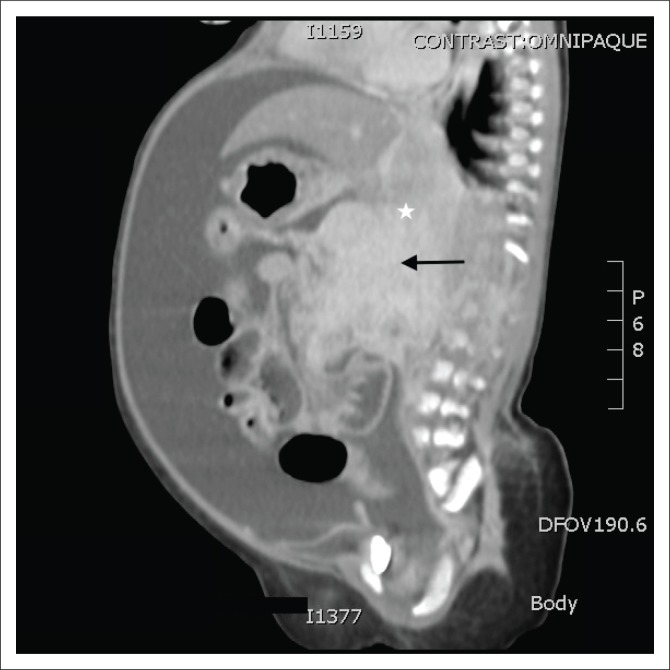
Sagittal post-contrast computed tomography image illustrating a bulky, inhomogeneously enhancing mass in the head of the pancreas (black arrow) with ill-defined margins and extension into the porta hepatis (white star).

**FIGURE 3:**
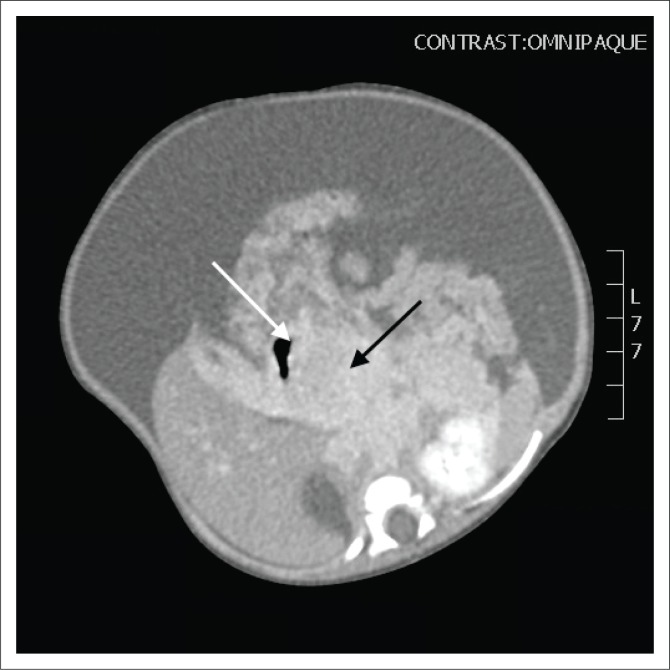
Axial post-contrast computed tomography image demonstrating the locally invasive pancreatic mass (black arrow) with a poor plane of separation from the D2 segment of the duodenum (white arrow).

This was followed by a computed tomography (CT) scan of the abdomen and pelvis with an intravenous and oral contrast agent. Computed tomography imaging showed an ill-defined, enhancing mass in the head of the pancreas (Figures 1–3), with a poor plane of separation from the D2 segment of the duodenum (Figures 1 and 3). Enhancing soft tissue, which extended beyond the confines of the pancreatic head into the region of the porta hepatis, was also noted and appeared to be contiguous with this mass (Figure 1).

**FIGURE 4:**
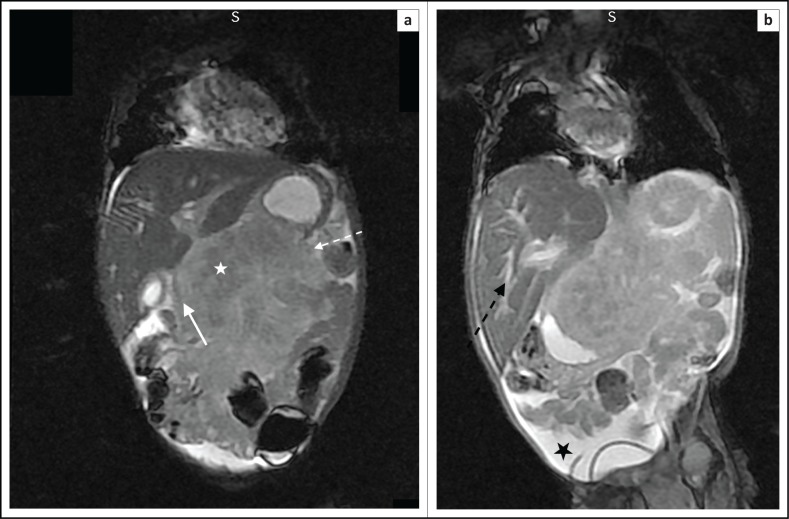
Coronal T2-weighted (T2W) magnetic resonance images: (a) Pancreatic mass (white star) with ill-defined margins and a lobulated contour (broken white arrow). Evidence of peripancreatic extension to the porta hepatis. This lesion is locally invasive, as demonstrated by the inseparability from the D2 segment of the duodenum (solid white arrow); (b) Associated findings of dilated intrahepatic bile ducts (broken black arrow) and ascites (black star).

**FIGURE 5:**
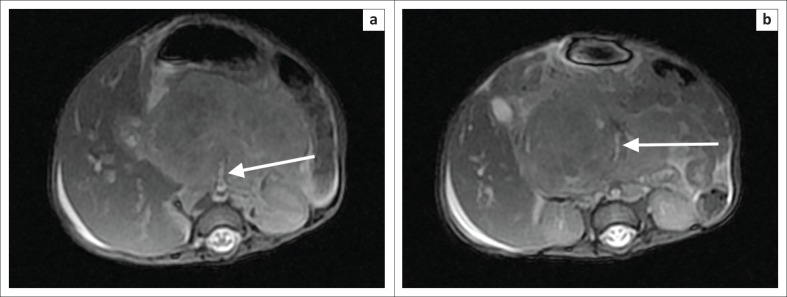
Axial T2-weighted (T2W) magnetic resonance images showing the pancreatic head mass with intermediate signal intensity and encasement of the superior mesenteric artery (white arrow) in (a) and (b) – sandwich sign.

The parents of the child decided to use a traditional healer, which delayed the magnetic resonance imaging (MRI) of the abdomen by 2 months. The MRI abdomen demonstrated a pancreatic mass of poorly defined margins that had progressively enlarged and extended across tissue planes (Figure 4a and b). There was encasement of the superior mesenteric artery as demonstrated by the sandwich sign (Figure 5a and b), peripancreatic extension into the porta hepatis (Figure 4a) and also intrahepatic bile duct dilatation (Figure 4b).

The differential diagnosis based on the imaging studies was that of a pancreatic vascular neoplasm; however, with the laboratory evidence of Kasabach–Merritt phenomenon (KMP), this prompted the diagnosis of a pancreatic KHE. Given the large size and peripancreatic extension of this lesion, a differential diagnosis of pancreaticoblastoma was considered; however, these lesions are typically heterogeneous in appearance with both cystic and solid components and often have calcifications.

**FIGURE 6:**
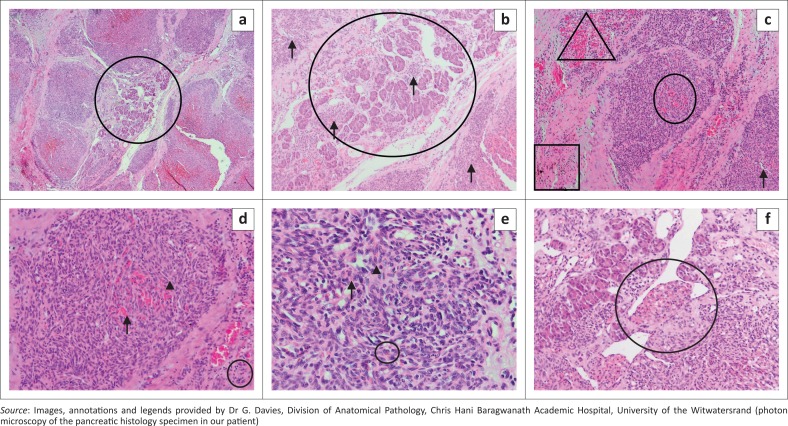
Photon microscopy of the pancreatic histology specimen with different magnification: (a) this low power image shows residual pancreatic tissue (centre circle) with surrounding lobules of the neoplastic infiltrate (4 × 10 magnification); (b) This higher power view of the same area (a) shows pancreatic acina, with surrounding and intermingled lobules of the neoplastic infiltrate (arrows) (10×10 magnification); (c) The neoplasm comprises spindled cells in swirling nodules. Slit-like vascular spaces are present (arrow). Extravasated red blood cells (oval) and hyaline globules (triangle) can be seen. Haemosiderin pigment is noted (square) (10×10 magnification); (d) This higher power view of the same area (c) better demonstrates the extravasated red blood cells (arrow) and hyaline globules (arrow head). Occasional inflammatory cells are visible (circle) (20×10 magnification); (e) This high-power image shows the spindle cell detail (40×10 magnification). The nuclei are elongated and bland with little pleomorphism and pale cytoplasm, with indistinguishable cell borders (arrow head). Haemosiderin pigment is evident (circle) and occasional extravasated red blood cells are present (arrow); (f) This image shows pancreatic acina and adjacent tumour. In the centre is a nodule of tumour bulging into a vessel, the so-called ‘promontory sign’ (circle) (20×10 magnification).

After correction of the patient’s thrombocytopenia, an open biopsy of the pancreatic mass was taken at laparotomy. Macroscopy of the specimen demonstrated a dark brown wedge of tissue measuring 10 mm × 5 mm× 6 mm. Microscopy (Figure 6a–f) showed representation of pancreatic parenchyma, which contained a lobulated spindle cell neoplasm with slit-like vascular spaces, extravasated red blood cells, haemosiderin and hyaline globules. There was no necrosis and mitotic activity was 0 in 10 consecutive high-power fields (HPFs). No plasma cell infiltrate, no gland or acinus formation and no squamous corpuscles or nests were noted. In the presence of adequate controls, the following immunohistochemical stains were performed: cytokeratin AE1/3 negative, strongly positive CD34 and negative human herpes virus 8 (HH8). Based on the above findings, the anatomical pathology report was indicative of a KHE.

Although the ascites had improved, the jaundice and thrombocytopenia had become progressively worse. The patient was treated with interferon alpha but subsequently demised.

## Discussion

In approximately 70% of cases, KHE may be complicated by KMP, a life-threatening complication characterised by profound thrombocytopenia and consumptive coagulopathy.^1^ This is secondary to the activation and aggregation of platelets, which results in thrombocytopenia, consumption of fibrinogen and ongoing fibrinolysis, which ultimately leads to tumour enlargement with intralesional bleeding. Kasabach–Merritt phenomenon is typically associated with larger, more aggressive lesions and there are currently no specific criteria to risk-stratify patients with regard to the occurrence or recurrence of KMP.^3^ Cutaneous lesions with a maximal diameter greater than 8 cm, or lesions with involvement of the retroperitoneum or visceral organs, have been implicated as risk factors.^3^ Despite KMP traditionally being described as thrombocytopenia or coagulopathy associated with any vascular anomaly, it is not a complication of infantile or congenital haemangioma and is a common occurrence with KHE.^5^

Haemangiomas may appear similar to KHE on imaging, except for the more aggressive and locally invasive nature of this lesion.^6^ This together with laboratory evidence of KMP may assist in the differentiation between these two entities.

On ultrasound, KHE appears as a homogeneous, ill-defined, soft tissue mass, which may be isoechoeic or hyperechoic compared to the pancreas, with associated mild to marked increased vascularity on colour Doppler.^2,6^ All previously reported cases described a mass in the head of the pancreas, as in this case report.^6^ Although the possibility of a vascular tumour could be suggested on US, a more specific diagnosis cannot be made because of the limitations in demonstrating the infiltrative portions and exact extent of KHE. Computed tomography and MRI together with magnetic resonance cholangiopancreatography (MRCP) are of added value in localising the mass, assessing the tumour extent and giving information of any associated findings such as dilation of the pancreatic and biliary ducts, duodenal obstruction and ascites. Leung et al. presented a case of a pancreatic KHE presenting with neonatal duodenal obstruction.^1^

The infiltrative nature of these lesions has been reported as a unique characteristic of KHE, assisting in differentiating this from other vascular tumours, with the exception of angiosarcomas.^2^ MRI findings typically show a lobulated, invasive mass with increased signal intensity on T2WI and avid enhancement following intravenous gadolinium.^6^ Ryu et al. reported 12 cases of pathologically proven KHE across the body, with lesions demonstrating heterogeneous regions of isointensity or mild hyperintensity on T2 weighted imaging (T2WI) and heterogeneous enhancement post-contrast administration.^2^ Regions of low signal on T2WI are likely secondary to hemosiderin deposition and dense hyaline stromal responses.^2^ The heterogeneous enhancement was likely associated with the pathological nature of vascular channels and thin-walled lymphatic vessels in KHE.^2^

The treatment of pancreatic KHE is variable and depends on associated vascular invasion, extent of the mass and the degree of biliary obstruction.^6^ Medical therapies for KHE and KMP include systemic steroids, vincristine, inteferon-alpha and aspirin.^7^ In cases with associated biliary obstruction, complete surgical resection with a Whipple operation has been reported and there have also been successful attempts at biliary stenting and external drainage (palliative surgery), pending spontaneous tumour regression.^1,6^ Historically, angiography and embolisation have been used for the diagnosis and treatment of KHE.^8^ These procedures are currently primarily used as an adjunct to planned surgical resection to minimise bleeding intra-operatively.^8^

Mammalian target of rapamycin (mTOR) inhibitors such as sirolimus have proven to be effective in the treatment of cutaneous KHE.^7^ Triana et al. described a case of pancreatic KHE not responding to sirolimus and suggested the possibility of a different molecular profile compared to the cutaneous form as a result of the resistance to mTOR inhibitors treatment.^7^

## Conclusion

Pancreatic KHE is an exceedingly rare vascular tumour that mostly occurs during infancy and early childhood. KHE may be misdiagnosed on imaging as a congenital or infantile haemangioma; however, the infiltrative pattern of KHE with its ill-defined margins and extension across tissue planes is a unique feature of KHE and may assist in the differentiation. The presence of KMP is useful in supporting the specific diagnosis. Despite KMP traditionally being described as thrombocytopenia or coagulopathy associated with any vascular anomaly, it is not a complication of infantile or congenital haemangioma. Although imaging modalities such as CT and MRI may assist in delineating this lesion, the formation of a clear pre-operative diagnosis remains difficult and early surgical exploration and rapid frozen biopsy are recommended. Pancreatic vascular malformations are best managed in a multidisciplinary setting with extensive experience in the treatment of vascular anomalies, as the treatment differs based on the size, location, associations and complications.
